# Blood-Biomarkers for Glucose Metabolism in Preterm Infants

**DOI:** 10.3390/biomedicines11092377

**Published:** 2023-08-24

**Authors:** Mia O. Bjerager, Bo M. Hansen, Frederik Sørensen, Jes R. Petersen, Kristian V. Jensen, Brian R. Hjelvang, Anna C. Hvelplund, Dorte A. Olsen, Aneta A. Nielsen, Julie L. Forman, Ivan Brandslund, Gorm Greisen, Carina Slidsborg

**Affiliations:** 1 Department of Neonatology, Nordsjælland Hospital, 3400 Hillerød, Denmark; mia.ortved.bjerager@regionh.dk (M.O.B.); bo.moelholm.hansen@regionh.dk (B.M.H.); 2 Department of Public Health, Section of of Biostatistics, University of Copenhagen, 1352 Copenhagen, Denmark; soerensen.freddie@gmail.com (F.S.); jufo@sund.ku.dk (J.L.F.); 3 Department of Neonatology, Herlev Hospital, 2730 Herlev, Denmark; jes.reinholdt@dadlnet.dk; 4 Department of Neonatology, Hvidovre Hospital, 2650 Hvidovre, Denmark; ktij@regionsjaelland.dk (K.V.J.); brian.rafn.hjelvang.01@regionh.dk (B.R.H.); anna.carolina.hvelplund@regionh.dk (A.C.H.); 5 Department of Biochemistry and Immunology, Vejle Sygehus, University of Southern, 7100 Vejle, Denmark; dorte.aalund.olsen@rsyd.dk (D.A.O.); aneta.aleksandra.nielsen@rsyd.dk (A.A.N.); ivan.brandslund@rsyd.dk (I.B.); 6 Department of Neonatology, Copenhagen University Hospital—Rigshospitalet, 2100 Copenhagen, Denmark; gorm.greisen@regionh.dk; 7 Department of Ophthalmology, Copenhagen University Hospital—Rigshospitalet, 2100 Copenhagen, Denmark

**Keywords:** preterm infants, blood biomarkers, glucose, metabolism, plasma-protein depletion, healthy reference levels

## Abstract

This was an exploratory, prospective, longitudinal, cohort study that aimed to establish “healthy” reference levels related to growth parameters and glucose metabolites in preterm infants. This was conducted to further investigate growth and metabolic disturbances potentially related to neonatal illness. The study sample consisted of 108 preterm infants born before 32 weeks in 2018–2019 in the Capital Region of Denmark. Repetitive blood samples were acquired at the neonatal wards, while clinical data were obtained from the regional hospital medical record system. Thirty-four “healthy” preterm infants (31%) were identified. The “ill” infants were divided into four subgroups dependent on gestational age and small for gestational age. Reference levels for the growth parameters and metabolic biomarkers glucose, albumin, and adiponectin, and two glucose control indicators, glycated albumin and fructosamine, were determined for the “healthy” and “ill” subgroups. The “ill” extremely preterm infants had increased glucose levels (mean difference 0.71 mmol/L, 95% CI 0.23; 1.18 mmol/L) and glycated albumin (corrected; %) (mean difference 0.92 mmol/L, 95% CI 0.38 mmol/L;1.47 mmol/L) compared to the “healthy” infants. In “ill” extremely preterm infants and “ill” very preterm infants born small for gestational age, levels of biomarkers containing proteins were decreased. In the “Ill” extremely preterm infants and infants born small for gestational age, postnatal growth was continuously decreased throughout the postconceptional period. The short-term glucose-control indicator, glycated albumin (corrected; %), reflected well the high glucose levels due to its correction for the depleted plasma-protein pool.

## 1. Introduction

Preterm infants born before 32 gestational weeks are in a nutritional emergency state after birth [1]. Their immature gastrointestinal tract does not support full enteral feeding, so intravenous glucose is usually given to prevent hypoglycemic events [2]. Additionally, extremely preterm infants are given parenteral nutrition with amino acids and lipid to support growth and optimize long-term neurodevelopmental outcomes [1]. Early intravenous infusion of glucose and prolonged parenteral nutrition may exacerbate metabolic derangements due to the immature organ system’s impaired glucose homeostasis [3]. The immature liver may catabolize body proteins to generate more glucose [4,5,6,7,8]. The immature pancreatic production of ineffective proinsulin and peripheral insulin resistance leads to ongoing glucose production, which possibly worsens already existing metabolic derangements [9]. Many preterm infants are found to be hyperglycemic during the early postnatal period [8,10,11,12]. In addition, prolonged hyperglycemia is associated with increased neonatal morbidity, mortality, and long-term unfavorable health outcomes in preterm infants [8,10,11,12]. Whether hyperglycemia plays a causal role or merely reflects a stress response related to neonatal morbidity remains unclear [8,13]. In preterm infants, intrauterine growth restriction could be associated with generation of greater metabolic derangements [4]. In addition, combined growth restriction and metabolic derangements potentially alter the infants’ cell programming, leading to cardiovascular diseases and type-2 diabetes later in life [1,14,15,16].

Although the literature on neonatal growth and metabolism is substantial, limited knowledge exists on the complex interplay between being born premature, intrauterin growth restriction, postnatal growth pattern, and related metabolic blood biomarker levels during the neonatal period, which potentially includes aggravation of biomarker and growth disturbances in infants having neonatal morbidities.

The aim of this exploratory longitudinal cohort study was to investigate these interconnections and the related neonatal outcomes in more detail. In order to do this we herein define some clinical criteria to select “healthy” infants to determine “healthy” reference levels related to postnatal growth and some metabolic biomarkers, and two glucose control indicators relevant for diabetes mellitus [17,18]. Herein, we show that “ill” extremely preterm infants, or “ill” preterm infants born small for gestational age, present significantly increased growth and metabolic disturbances compared to infants considered “healthy”. The glucose control indicator glycated albumin (corrected, %), when corrected for albumin levels, appeared to be a valid biomarker for monitoring chronic glucose levels in preterm infants, including “ill” infants whose plasma-protein levels are potentially depleted.

## 2. Materials and Methods

This was an explorative study designed as a population-based, prospective, longitudinal, multicenter study and was conducted in the period 2018–2019 in the Capital Region of Denmark.

### 2.1. Identification and Recruitment of the Infants

Local physicians responsible for four different neonatal departments recruited preterm infants born at a gestational age (GA) less than 32 weeks. The hospital electronic health record system (EPIC) applied in the four regional hospitals provided patient information for identification of infants. A total of 108 preterm infants were recruited.

### 2.2. Sample Procedures

Blood samples were collected (mostly capillary blood by heel prick) from the 14th postnatal day after birth and approximately every two weeks until term. Collection, preparation, and storage at the clinical biochemistry departments was standardized across the four hospitals. The collected blood volume depended on the infants’ body weight: (1) <1 kg had 0.6 mL. taken, while (2) 1 kg or more had 1.0 mL taken. Samples consisted of capillary blood retrieved from infant heel prick and, more rarely, venous specimens. Samples were collected in standard tubes (Hettich Katalog nr. 450551 Lithium Heparin). Sample were centrifuged at 3000× *g* in 10 min at room temperature within 2 h of sample collection and stored in cryotubes in a −80 °C freezer, or sometimes initially for 2–3 months in a −60 °C freezer.

### 2.3. Target Analytes and Measurements

For analyses of glucose (hexokinase method, Roche Diagnostics, Basel, Switzerland), fructosamine (colorimetric method, Roche Diagnostics, Basel, Switzerland), and glycated albumin and total albumin (enzymatic method, quantILab, Werfen, Milano, Italy), samples were thawed on a heat block for approximately 10 min, mixed gently using a vortex, and spun breifly. Samples lacking material (i.e., less than 100 µL) for analysis were diluted 2-fold with physiological saline (0.9% NaCl). Analyses were performed on Cobas8000 c702 and c502 modules (Roche Diagnostics). Analytical coefficient of variation (CV) was for glucose <2%, fructosamine <2.3%, and glycated albumin <3%. Glycated albumin (corrected; %) was calculated according to the kit insert. Fructosamine (corrected value; corr) was obtained as (fructosamine/albumin) * mean (healthy) albumin concentration.

A commercially available ELISA kit (TECOmedical, Sissach, Switzerland) was used to quantify adiponectin in the samples according to the manufacturer’s procedure. The samples were diluted 1200-fold. Three assay quality controls, two in the kit and one in-house, were included in each run to evaluate assay performance. CV for adiponectin < 18%.

### 2.4. Patient Data Retrieval and Definitions

Clinical data were extracted from the hospital electronic record system and cross-checked with individual patient records for inconsistencies. The retrieved data were GA (days), birth weight (BW; grams), head circumference (head circ; cm), length (cm), body weight (grams), glucose measured repeatedly in the blood, parenteral glucose (mg/day), parenteral nutrition (kcal/day), and neonatal surgery codes and diagnoses. Before analysis of data, based on neonatal clinical criteria expected to influence glucose metabolism in preterm infants, the study sample was divided into “healthy” and “ill” infants. The criteria for “ill” were: (1) born SGA, (2) in need of glucose infusion, parenteral nutrition, or antibiotics for more than 7 days during the entire neonatal period, or (3) a neonatal diagnosis of: (3a) ductus arteriosus persistence (DAP) defined as clinically significant by the neonatologist, (3b) intraventricular hemorrhage (IVH; stage 1–4), (3c) necroticizing enterolitis (NEC)—any stage, (3d) brochopulmonal dysplasia (BPD) defined as need of respiratory support (i.e., mechanical ventilation, CPAP, Opti-flow) for at least 28 days, (3e) retinopathy of prematurity (ROP)—any stage, or (3f) any major congenital malformation. SGA was defined as BW-2SD relative to mean BW expected for a given GA [19]. The one “ill” LGA infant was pooled with the AGA preterm population. Thus, all the remaining infants were labeled appropriate-/large for GA (A-/LGA). The “ill” group was further divided accoring to prematurity and born SGA.

### 2.5. Definition of “Healthy” and “Ill” Preterm (at Least One Clinical Criteria Fulfilled) Subgroups

1. “Healthy” infants, born GA < 32 weeks, AGA.
2. “Ill” very preterm infants, born GA ≥ 28 weeks, A-/LGA.
3. “Ill” very preterm infants, born GA ≥ 28 weeks, SGA.
4. “Ill” extremely preterm infants, born GA < 28 weeks, A-/LGA.
5. “Ill” extremely preterm infants, born GA < 28 weeks, SGA.

### 2.6. Ethics Statement

The present study was approved by the Danish Ethics Committee (H-17034984) and the Data Protection Agency. Informed consent was obtained from all individuals included in this study and/or their legal guardian(s).

### 2.7. Statistical Analysis

Descriptive data are presented as mean +/-SD and number (%). Growth curves and blood biomarkers are visualized in spaghetti plots. Differences between the “ill” and “healthy” groups were estimated using a generalized additive model including a spline-effect of postconceptional age (PCA) to account for a potentially non-linear effect and a random effect of the infant to account for repeated measurements on each infant. The inter- and intraindividual variation in the blood biomarkers was assessed by normal ranges and intraclass correlation coefficients (ICCs). The associations of fructosamine and glycated albumin were investigated with running averages of all blood glucose measurements (recorded in the hospital electronic record system) using a linear mixed model with a fixed effect of the running average and a random effect of the infant. All *p*-values were adjusted in multiple testing using the method of Benjamini and Hochberg, which controls the false discovery rate [20]. An adjusted *p*-value < 0.05 was considered statistically significant. Analyses were performed with R version 4.0.3 (R Foundation for Statistical Computing, Vienna, Austria) [21].

## 3. Results

**Figure 1:**
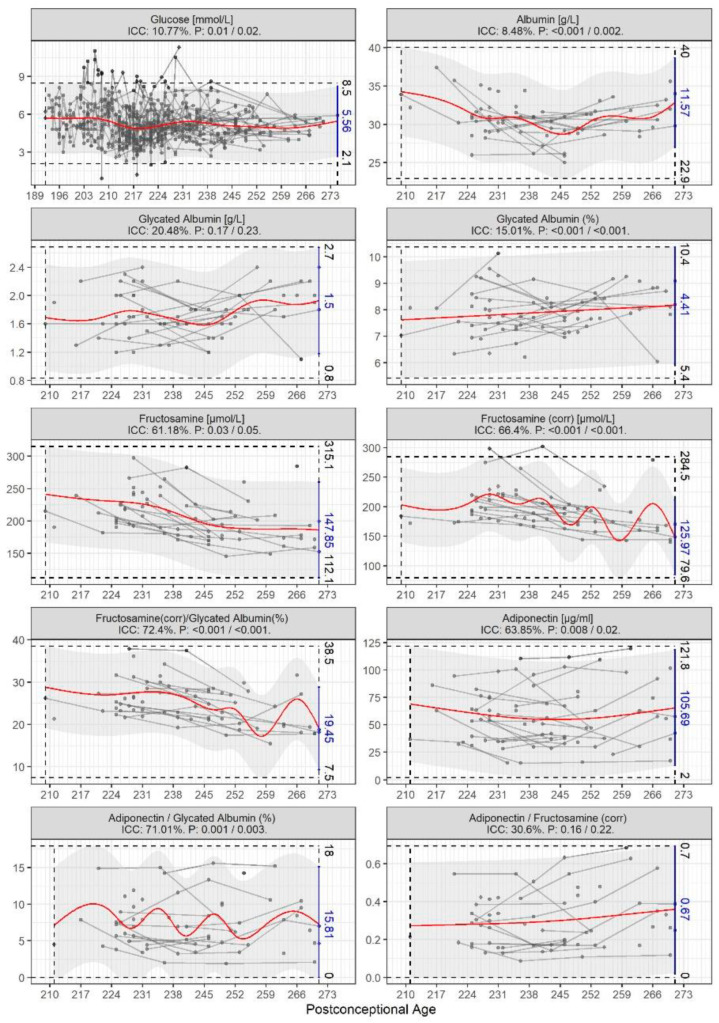
Blood glucose biomarkers from “healthy” children born GA < 32 weeks, AGA with no clinical signs of neonatal morbidities across the postconceptional period. Estimated mean and normal ranges based on the generalized additive model are shown as solid lines for each infant and gray shade with minimum and maximum values indicated by dashed lines. The red line represents the estimated mean of the “healthy” reference values. ICC = intraclass correlation.

**Figure 2:**
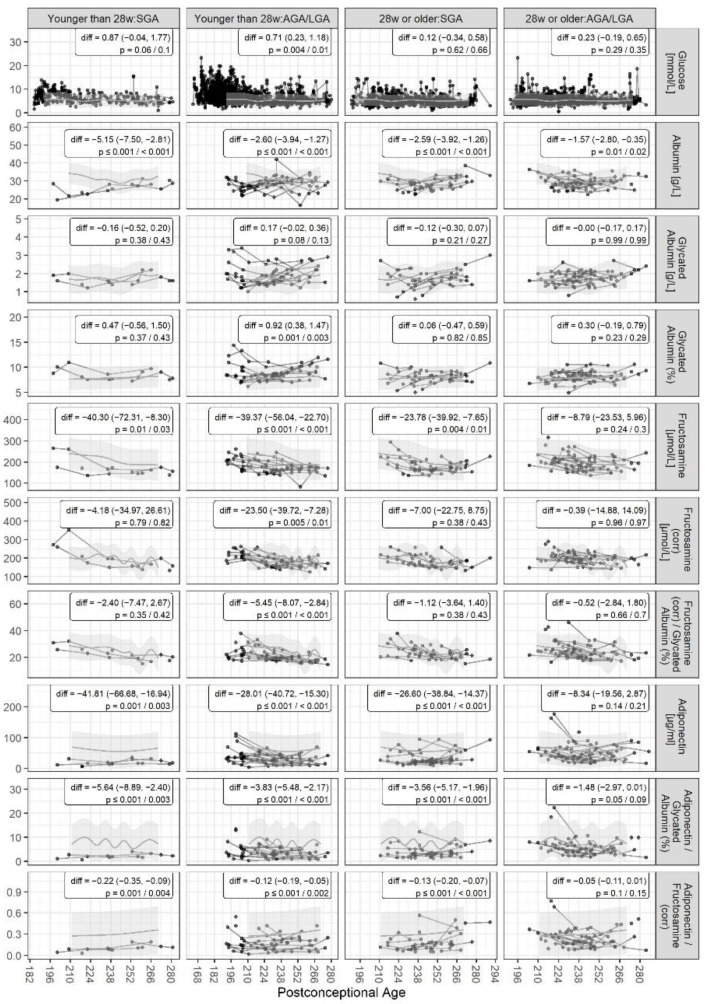
Blood biomarkers and related ratios in “ill” subgroups divided according to gestational age, born SGA vs. A-/LGA across the postconceptional period. For comparison, normal ranges from the “healthy” subgroup are indicated in grey shading. Diff is the estimated mean difference compared to the “healthy” subgroup with 95% CI and *p*-values (unadjusted/adjusted) based on the generalized additive model.

The study population consisted of a total of 108 preterm infants born between GA 23 + 3 and 31 + 6 weeks with a BW from 565 to 2645 g. Infants born SGA had GA 26 + 2 to 31 + 6 weeks and BW from 565 to 1342 g. Detailed characteristics of “healthy” and four “ill” preterm subgroups are presented in Table 1. The “healthy” subgroup consisted of 34 (31%) infants of which 16 (47%) were male. The “ill” subgroups showed a progressive association between increase in prematurity and occurence of neonatal morbidity (i.e., ROP, IVH, NEC, DAP, and/or BPD). Neonatal morbidity occurred more frequently in infants with GA < 28 weeks and/or SGA, and in the male gender. The time evolution of the metabolic biomarkers across the postconceptional period is presented in Figure 1 and Figure 2. Data from the “healthy” subgroup of infants is shown in Figure 1 together with estimated mean, normal range, and intraclass correlation (ICC). All biomarkers, except for glycated albumin (g/L) and the ratio of adiponectin/fructosamine, showed a significant association with postconceptual age. Similar to a study on adults, an approximately linear association was found between glycated albumin (corrected, %) and log-transformed postconceptional age, estimated by the equation [Gla (%) = 4.98 ∗ log (PCA) − 3.96] [22]. Individual levels of fructosamine, fructosamine (corr), fructosamine (corr)/glycated albumin (corrected, %), adiponectin, and adiponectin/glycated albumin (corrected, %) appeared to be fairly stable, with ICCs of 61.18%, 66.4%, 72.4%, 63.85%, and 71.01%, respectively. The remaining blood biomarkers had low ICCs.

Blood biomarker levels of the four “ill” subgroups, and statistical results when comparing them to the “healthy” subgroup, are presented in Figure 2. Infants with GA < 28 weeks and A-/LGA had significantly elevated glucose and glycated albumin (corrected, %) levels compared to the “healthy” infants, and decreased levels of remaining potein-containing biomarkers, except glycated albumin (g/L). Estimated differences in glucose and glycated albumin (corrected, %) levels between the infants with GA < 28 weeks born SGA or A-/LGA, compared to the healthy subgroup, were almost similar, although results for those born SGA were statistically insignificant. The remaining “ill” subgroups showed trends towards higher blood glucose and glycated albumin (corrected, %) levels. Infants born at GA ≥ 28 and SGA had significantly lower levels of albumin, fructosamine, adiponectin, adiponectin/glycated albumin (%) ratio, and adiponenctin/fructosamine (corr) ratio, compared to “healthy” infants. Infants with GA ≥ 28 weeks and A-/LGA had significantly lower albumin levels.

**Figure 3:**
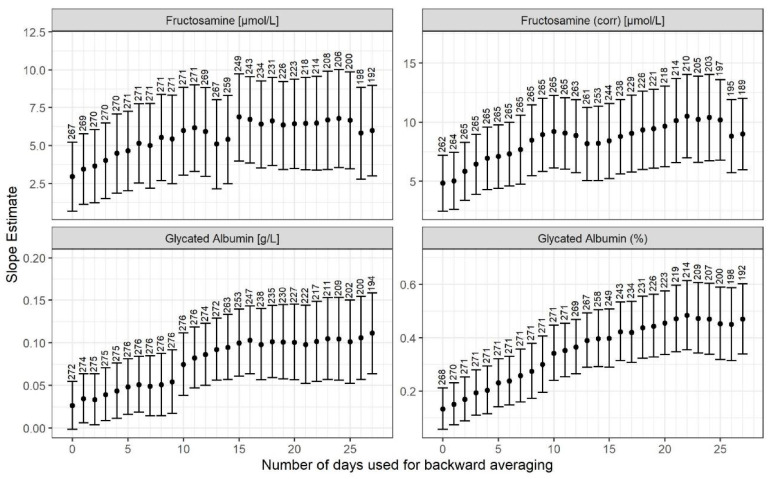
The estimated associations between levels of glycated albumin (%), glycated albumin, fructosamine (corr), and fructosamine with backwards-running averages of glucose measurements; the strongest association for corrected glycated albumin (%) was found on the 22nd postnatal day. Valid interpretation of associations for the other potential glucose control indicators were difficult due to a depleted total plasma-protein pool and insufficient correction parameters.

Figure 3 shows the estimated associations between levels of glycated albumin (corrected, %), glycated albumin, fructosamine (corr), and fructosamine and postnatal age with backwards-running averages of glucose measurements. The strongest association for glycated albumin (corrected,%) was found on the 22nd postnatal day. Valid interpretation of associations for the other potential glucose control indicators mentioned were not possible due to a depleted total plasma-protein pool, with insufficient adjustment for this.

**Figure 4:**
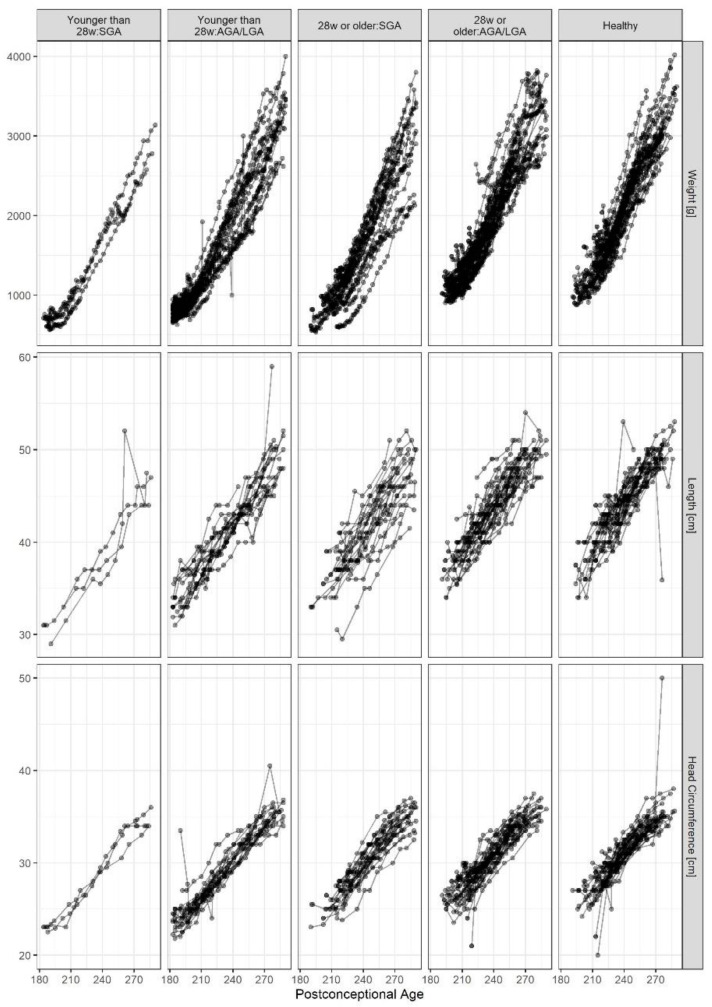
Growth curves for body weight, body length, and head circumference in subgroups of preterm infants across the postconceptional period. Infants born extremely premature, small for gestational age, and experiencing neonatal morbidities remain more growth-restricted throughout the neonatal period.

Growth curves of length, body weight, and head circumference are presented in Figure 4. Postnatal growth was subnormal throughout the postconceptional period in “ill” preterm infants born GA < 28 and/or SGA regarding body weights, length, and head circumference.

## 4. Discussion

There is limited knowledge about metabolic blood biomarkers in preterm infants and their association with prematurity, growth retardation, and neonatal morbidities [23]. This is likely due to the inherent heterogenity of these factors in the preterm population, which makes it difficult to separate causes and effects [23]. Herein, we found a clear associaton between neonatal morbidity, increasing prematurity, SGA, and postnatal growth restriction. Similarly, each of these factors was associated with increasing metabolic biomarker derangements.

Based on clinical conditions known to influence glucose metabolism, the study cohort was divided into a “healthy” (reference) group and four “ill” subgroups related to degree of prematurity and presence of SGA [3,5,8,10,11,12,24]. Defining a “heathy” reference group is a novel apporach. This allowed us to suggest “healthy” reference values for both our chosen metabolic biomarkers and growth parameters. It was reasuring for the validity of our neonatal clinical criteria that the “healthy” glucose range that was generated corresponded well with the “normal” glucose range internationally agreed upon for preterm infants [24]. This suggests that our clinical criteria are appropriate for the intended purpose of the study.

Herein, we investigated some essential metabolic biomarkers for diabetes mellitus, namely glucose, adiponectin, and albumin. We found that the glucose levels were generally higher in all the “ill” preterm subgroups, especially during the early postconceptional period. Similarily, several previous studies found that hyperglycemia was especially pronounced during the early postconceptional period [3,8,11,12,24,25]. As was previously described, we found a clear association between prolonged hyperglycemia and increased occurrence of neonatal morbidity (such as IVH, NEC, ROP) [8,10,11,12,26]. Previous studies also suggested that prolonged hyperglycemia was associated with a higher mortality rate, and long-term unfavorable health outcomes, such as such as cardiovascular diseases and type-2 diabetes [8,10,11,12,26].

Herein, we also investigated the use of two less commonly used GCIs, glycated albumin and fructosamine, as potential candidates to measure the burden of “chronic” high glucose levels in the preterm cohort. In the “ill” preterm infants, the protein-containing biomarker levels were reduced, which could potentially influence values of the GCIs under investigation. Herein, both GCIs had shorter half-lives than that of the golden standard biomarker, HbA1c, which is normally used for monitoring long-term glucose levels in diabetic patients. The shorter half-lifes of the new GCIs makes them more relevant to use in preterm infants, especially during the first months after birth [27]. Use of the standard Hb1Ac as a GCI in studies on preterm infants would be invalid due to the high levels of Hemoglobin F in these infants, and its long half-life of 120 days, and because it also shows values that are strongly influenced by maternal glucose levels.

In the “ill” preterm infants, we needed to compensate for the depleated plasma-protein pool, so we adjusted both of the GCIs for the reduced albumin levels. This worked well for glycated albumin as the corrected value reflected well the hyperglycemic state in the “ill” extremely preterm infants and infants born at A-/LGA. This means that glycated albumin (corrected, %) could be a valid biomarker for monitoring short-term glucose levels in “ill” preterm infants suffering from plasma-protein depletion [28]. For fructosamine, correction for plasma-protein levels did not work well. This biomarker would have benefitted from being corrected for the total plasma-protein pool instead of albumin, and likely other confounders as well [29,30,31].

The reduced levels of plasma proteins that we encountered in this study could be a direct consequence of the immature liver attempting to generate more glucose by catabolizing plasma proteins to counteract the inefficient uptake of glucose in the periphery tissues [4,5,6,7,8]. We speculate whether the total body protein pool in these “ill” preterm infants was depleted, which could also prohibit optimal postnatal growth [32,33]. In fact, we did find that the “ill” infants had suboptimal postnatal growth compared to “healthy” infants [3,5,8,10,11,12,24]. In addition, growth restriction was more pronounced in “ill” infants born extremely premature or SGA, and was also consistent with decreased protein-containing biomarker levels [4].

A previous study suggested that infants born SGA are already depleted in their total body protein pool at birth [28]. Although we found significantly reduced protein-containing biomarker levels in the “ill” infants born SGA, we found no significant increase in glucose levels, which was most likely related to the small sample size [4]. The overall energy demands in infants born SGA may be increased, which could be attributed to the prononced continous postnatal growth restriction seen. It is well-known that children born with intrauterin growth restriction are at increased risk of metabolic diseases such as developing type-2 diabetes later in life [15,16].

The protein hormone adiponectin that is known to regulate body fat and glucose homeostasis through the enhancement of insulin sensitivity was lower in the circulation of the “ill” preterm infants compared to that of the “healthy” infants. This could be related to the plasma-protein depletion that was found in the “ill” infants in general. In previous studies, adiponectin was shown to be inversely related to glucose level in the circulation, and was dependent on both body weight and growth retardation at birth [34,35].

### Strengths and Limitations

A strength of this study is the prospective longitudinel design, which allowed us to investigate blood biomarkers and postnatal growth restriction in a population-based sample of preterm infants born in a large geographic area (the Capital Region, Denmark) and during the entire neonatal period. We obtained a sample that was large enough to demonstrate significant differences in metabolic biomarker levels for varying degrees of prematurity and for infants born SGA vs. A-/LGA between “healthy” and “ill” preterm subgroups. In this exploratory study, via the use of neonatal clinical criteria, “healthy” preterm infants were determined to identify “healthy” reference ranges for growth parameters and our chosen blood biomarkers. In future large-scale datasets, the next step would be to investigate whether these biomarkers and other new biomarkers can predict development of neonatal diseases, such as ROP. A recent study suggested that glucose and adiponectin could be key players in the ROP pathogenesis (Fu et al., 2018 [36]).

This study also have some limitations that should be mentioned. As preterm infants are in an unnatural state outside the mother’s womb, the concepts of “normality” are controversial. In this study, we acknowledge the inherent difficulty in declaring absolute “normality” and labeling the control group “healthy” instead. Collinearity makes it difficult to separate the effects of neonatal morbidities, prematurity, and growth restriction; thus, residual confounding cannot be ruled out. For the “ill” infants, prematurity, neonatal morbidity, and parenteral nutrition—including glucose infusion—could each have contributed to the high glucose levels, and therefore the effect of each contributing factor is difficult to quantify. The consideration of maternal risk factors was outside the scope of this study, so we cannot exclude that a part of the metabolic disturbances seen in some preferably “ill” preterm infants was related to such factors. Gestational diabetes is such a known risk factor, and could have contributed to glucose derangements. Previous studies found that preterm infants born from mothers with gestational diabetes could suffer from hyperglycemia related to LGA, macrosomia, excessive fetal growth, and several neonatal morbidities (Preda et al., 2021 [37]). In this study, as the “ill” preterm sample contained merely one LGA infant, and as the dominant feature was growth retardation rather than excessive fetal growth, it is unlikely that this risk factor contributed largely to our results. Our sample size did not allow us to investigate associations with the different neonatal morbidities, which were therefore pooled in spite of their obvious differences.

Finally, the blood specimens for glucose came from variable sources. Most of our blood glucose samples were capillary blood, instead of venous blood, so had measurements that were therefore lower than the actual values from venous samples. This possibly led to an underestimation of the actual glucose levels in the preterm infants of this study.

## 5. Conclusions

Herein, “ill” extremely preterm infants and preterm infants born SGA had continuous metabolic biomarker derangements and postnatal growth restriction during the neonatal period. In preterm infants, glycated albumin (corrected; %) represented hyperglycemia well and could be a valid short-term GCI for glucose monitoring in preterm infants. Future studies using large-scale datasets should be undertaken to validate the reference values generated from the novel neonatal clinical criteria and the heterogenicity of the human plasma proteome associated with maternal risk factors, prematurity, neonatal metabolism, neonatal morbidities, growth patterns, and the associated long-term unfavorable health outcomes.

## Figures and Tables

**Table 1:** Clinical characteristics of the four “ill” preterm groups and a “healthy” preterm group.

Variable	Ill Infants GA < 28 Wks SGA,	Ill Infants GA < 28 Wks AGA/LGA	Ill Infants GA ≥ 28 Wks SGA	Ill Infants GA ≥ 28 Wks AGA/LGA	Healthy Infants AGA
Group (*n*, %)	3 (3%)	18 (17%)	22 (20%)	31 (29%)	34 (31%)
Male (*n*, %)	0 (0%)	8 (44%)	16 (73%)	17 (55%)	16 (47%)
SGA (*n*, %)	3 (100%)	0 (0%)	22 (100%)	0 (0%)	0 (0%)
Gestational age (Median; Q1, Q3; wks.)	26.7 (26.5, 26.8)	25.8 (25.1, 26.4)	30.6 (28.9, 31.1)	29.1 (28.1, 30.5)	30.4 (29.0, 31.1)
Birth weight (Mean ± SD; gram)	642 ± 59	812 ± 133	1026 ± 221	1384 ± 341	1513 ± 272
Major neonatal morbidity: BPD/DAP/IVH/NEC/ROP [any, %]	2/1/0/0/2 [2, 67%]	8/12/3/4/9 [15, 83%]	1/3/1/1/4 [6, 27%]	4/6/4/1/7 [10, 32%]	0/0/0/0/0 [0, 0%]
Bronchopulmonary dysplasia [*n*, % of group]	2 [67%]	8 [44%]	1 [55%]	4 [13%]	0 [0, 0%]
Ductus arteriosus persistence [*n*, % of group]	1 [33%]	12 [67%]	3 [14%]	6 [19%]	0 [0, 0%]
Intraventricular hemorrhage [*n*, % of group]	0 [0%]	3 [17%]	1 [5%]	4 [13%]	0 [0, 0%]
Necrotizing enterocolitis [*n*, % of group]	0 [0%]	4 [22%]	1 [5%]	1 [3%]	0 [0%]
Retinopathy of prematurity [*n*, % of group]	2 [67%]	9 [50%]	4 [18%]	7 [23%]	0 [0, 0%]

Explanation of the rows: For morbidities: presented is total number of diagnoses given for subgroup, and in brackets [number of infants given at least one diagnosis, and % of group].

## Data Availability

The data presented in this study are available on request from the corresponding author. The data are not publicly available due to ethical restrictions.

## Abbreviations

Gestational age, GA; Birth weight, BW; Small for gestational age, SGA; Ductus arteriosus persistence, DAP; Intraventricular hemorrhage, IVH; Necroticizing enterolitis, NEC; Brochopulmonal dysplasia, BPD; Appropriate-/large for GA, A-/LGA; Postconceptional age, PCA.
